# Comparative analysis of clinical, electrocardiographic, angiographic and echocardiographic data of indigenous and non-indigenous residents of Yakutia with coronary artery atherosclerosis

**DOI:** 10.3402/ijch.v72i0.21219

**Published:** 2013-08-05

**Authors:** Natalya Vladimirovna Makharova, Michael Ivanovich Voevoda, Faina Fedorovna Lyutova, Irina Andreevna Pinigina, Vera Evstafievna Tarasova

**Affiliations:** 1 Yakutsk Scientific Center, SB RAMS, Yakutsk, Russia; 2 Institute of Internal Medicine, SB RAMS, Novosibirsk, Russia

**Keywords:** atherosclerosis, eccentric hypertrophy, indigenous population, non-indigenous population, Yakutia

## Abstract

**Aim:**

The aim of the study is to compare clinical, angiographic, electrocardiographic, echocardiographic data between indigenous and non-indigenous residents of Yakutia.

**Study design:**

We performed cross-sectional analysis of the Registry of Selective Coronary Angiography (SCAG) of the Yakutsk Republican Hospital for the period from 2004 to 2007. All patients (n=1,233) were admitted to hospital from all 35 regions of the Sakha Republic (Yakutia). Initially, 12 (1%) patients, who had abnormal coronary arteries and 259 (21%) patients with normal coronary arteries were excluded from this study. From the remaining 962 (78%) patients with detected coronary artery atherosclerosis 394 (41%) patients were excluded for having congenital heart malformations due to possible influence on the outcomes of examination for myocardial hypertrophy. Finally, only 568 patients were selected for further examinations.

**Methods:**

We analyzed clinical data, and the findings of selective angiography, multi-detector computed tomography (CT), electrocardiography (ECG), 24-hour Holter ECG monitoring and echocardiography.

**Results:**

(a) In the Sakha Republic (Yakutia) single-vessel coronary disease, coronary stenosis with 50–75% and 75–90% of constriction were detected more often among indigenous males, while multiple-vessel coronary stenosis was detected more often among non-indigenous males as well as stenosis with more than 90% of constriction. Lower calcium score mean (349.1±129.8 vs. 621.8±115.2) was observed among indigenous patients compared to non-indigenous patients; (b) Painless myocardial infarction, painless ischaemia, arterial hypertension and atrial fibrillation were detected more often among indigenous male compared to non-indigenous participants; (c) Based on the results of ECG and echocardiographic examinations, left ventricular (LV) hypertrophy, particular eccentric type of hypertrophy, was found more commonly among indigenous than non-indigenous males; and (d) By laboratory findings, indigenous males had significantly lower triglyceride levels, while platelet counts were higher compared to non-indigenous patients. Obesity was observed less frequently among indigenous men compared to non-indigenous men.

**Conclusion:**

The differences observed in this study are disputable and call for further studies. Collection of reliable data for women should be the aim of future studies.

Until now, quite a lot of epidemiological studies exploring region-specific risk factors for cardiovascular disease have been conducted in the Sakha Republic (Yakutia). Similar to morphological symptoms of atherosclerosis (1), risk factors for cardiovascular disease have been shown to be more prevalent among non-indigenous population of Yakutia (2–5).

## Materials and methods

We performed cross-sectional analysis of the Registry of Selective Coronary Angiography (SCAG) of the Yakutsk Republican Hospital for the period from 2004 to 2007. All patients (n=1,233) were admitted to hospital from all 35 regions of the Sakha Republic (Yakutia). Initially, 12 (1%) patients who had abnormal coronary arteries and 259 (21%) patients with normal coronary arteries were excluded from this study. From the remaining 962 (78%) patients with detected coronary artery atherosclerosis, 394 (41%) patients were excluded for having congenital heart malformations due to possible influence on the outcomes of examination for myocardial hypertrophy. Finally, only 568 patients were selected for further examinations. All subjects gave informed consent.

For the purpose of comparative study, patients with coronary artery atherosclerosis were divided into 2 groups (N=568):

 Group 1 included representatives of indigenous population (n=286), 266 of them were men (mean age: 54.2±0.5 years) and 20 were women (mean age: 55.0±1.6 years);

 Group 2 included representatives of non-indigenous population (n=282), 234 of them were men (mean age: 52.6±0.6 years) and 48 were women (mean age: 55.3±1.1 years).

Indigenous population included Yakuts, Evenks, Evens and Dolgans; non-indigenous population included Russians, Ukrainians, Byelorussians and others. Ethnicity of the patients was determined based on passport information (Table I).

**Table I:** Patient examination: methods and number of procedures

	Number of procedures (n=568)			
	
	Indigenous		Non-indigenous	
		
Patient examination methods	M	F	M	F
Clinical examination, smoking, body mass index	266	20	234	48
Total cholesterol, LDL, HDL, triglyceride levels	266	20	234	48
Haemostatic parameters (platelets, fibrinogens)	234	20	213	48
Selective coronary angiography (SCAG)	266	20	234	48
Multi-detector CT	26	10	37	12
Electrocardiography	266	20	234	48
Holter monitoring (ECG)	125	16	127	23
Echocardiography	202	20	189	48
Transoesophageal echocardiography (TEE)	80	50	10	8

LDL, low-density lipoprotein; HDL, high-density lipoprotein; CT, computed tomography; ECG, electrocardiography.

### Clinical examination

Clinical diagnosis of angina pectoris, arterial hypertension and chronic heart failure were established using angiographic, electrocardiographic, echocardiographic, laboratory examination methods in compliance with the Russian Society of Cardiology (RSC) guidelines.

Atrial fibrillation (AF) was determined based on documented clinical spontaneous AF episodes, findings of electrocardiography (ECG) and 24-hour Holter ECG monitoring. We used transoesophageal echocardiography (TEE) to detect left atrial appendage thrombi in patients with AF.

Type 2 diabetes mellitus was established based on documented regular medical records of the patients, who were followed-up on a regular basis in the Endocrinology Clinic (Dispensary).

### Functional examination methods

SCAG was performed using conventional Judkins method, and Axiom Artis BA angiography system (Siemens, Germany). We visually assessed types of coronary circulation (right coronary, left coronary or general) and number of diseased arteries (1-vessel, 2-vessel or 3-vessel involvement). Severity of stenosis was determined by means of quantitative coronary angiography, using following coronary angiographic classification: stage 1 – <50% stenosis; stage 2 – 50–75% stenosis; stage 3 – 75–90% stenosis; stage 4 – >90% stenosis.

Multi-detector CT was performed by using Somatom Sensation-4 scanner (Siemens) and included calculation of total calcium score. We used integrated software for automated quantitative analysis and employed conventional Agatston method (1990), which defined coronary calcification as an area with maximum density greater than 130 Hounsfield units (HU). Each calcified plaque in every slice was marked. Individual calcium scores for major coronary arteries and patient's total calcium score (expressed as the sum of calcium scores in all CT slices) were quantified automatically. Degree of calcification was expressed as total calcium score.

Standard 12-lead ECG was performed with the speed of 25 mm/s, using EK 512 equipment (Hellige). ECG results showing the presence of blocks, pacemaker, or Wolff-Parkinson-White syndrome (WPW) were excluded from the analysis.

The following voltage criteria for left ventricular (LV) hypertrophy were calculated: Sokolow-Lyon index (S_VI_+R_V5(V6)_), Gubner-Ungerleider criterion (R_I_+S_III_), Cornell voltage (R_aVL_+S_V3_), Cornell product [(R_aVL_+S_V3_+6 in females)]×QRS duration. The following were the signs of LV hypertrophy: R_aVL_ >11 mm, RV_5(V6)_ ≥27 mm, Sokolow-Lyon ≥35 mm, Gubner-Ungerleider >25 mm, Cornell voltage >28 mm in males and >20 mm in females, Cornell product >2,440 mm/ms (6, 7).

For 24-hour Holter ECG monitoring, patients were thoroughly instructed to accurately comply with the recommended monitoring protocol. Patients kept detailed record of activities and symptoms in their diaries throughout Holter monitoring period. Depression specific for ischaemia (horizontal or downsloping depression of ST segment for ≥0.08 s after the J point) or ST segment elevation of ≥1 mm persisting for ≥1 min with return to baseline for at least 1 min between the episodes was considered as an episode of ischaemia.

Echocardiography was performed using a conventional method. We used the following formula to calculate myocardial mass, as recommended by the American Society of Echocardiography (ASE): LV mass=0.8×(1.04[LVIDd+PWTd+SWTd)^3^ − (LVIDd)^3^])+0.6 g. All values were indexed to body surface area.

### Laboratory tests

Laboratory tests included analysis of platelet counts, levels of fibrinogen, glucose, total cholesterol and cholesterol fractions.

### Statistical analysis

Statistical analysis was done using standard SPSS software package (Version 11.5). The results were presented as M±m (M denotes arithmetic mean, m denotes standard deviation; 95% CI denotes 95% confidence interval). Normality of distribution of the quantitative variables was tested by Kolmogorov-Smirnov test. As the variables had mostly non-normal distribution, we employed non-parametric Mann-Whitney test to test statistical significance of the differences in median quantitative variables between 2 ethnic groups; qualitative variables were tested by Pearson's χ^2^-test for independent samples. Hypotheses were tested for 95% CI (p<0.05).

## Results

Clinical analysis showed that arterial hypertension (p=0.001), AF (p=0.047), left atrial appendage thrombi (p=0.025) and myocardial infarction without a previous history of angina pectoris (p=0.024) were found more often among indigenous males than non-indigenous. Patients had no differences in occurrence of myocardial infarction in previous histories, age at the time of past myocardial infarction, or in rates of type 2 diabetes mellitus (Table II).

**Table II:** Clinical characteristics of patients with coronary atherosclerosis, Yakutia

	Males			Females		
		
Clinical parameters	I n=266 (%)	NI n=234 (%)	p	I n=20 (%)	NI n=48 (%)	p
Age	54.2±0.5	52.6±0.6		55.0±1.6	55.3±1.1	
Smoking (%)	109 (41.0)	98 (41.9)	0.777	10 (50.0)	18 (37.5)	0.412
Obesity, BMI ≥30 kg/m^2^ (%)	82 (30.8)	92 (39.3)	0.047	4 (20.0)	32 (66.7)	0.001
Myocardial infarction (%)	167 (62.8)	145 (62.0)	0.851	14 (70.0)	34 (70.8)	0.945
Painless myocardial infarction (%)	141 (53.0)	99 (42.3)	0.024	4 (20.0)	11 (22.9)	0.846
Age at the time of myocardial infarction, M±m	51.2±0.6	50.6±0.6	0.269	48.3±1.6	52.9±0.8	0.010
Arterial hypertension (%)	245 (92.1)	187 (79.9)	0.001	20 (100.0	48 (100.0)	–
Atrial fibrillation (%)	75 (28.2)	48 (20.5)	0.047	5 (25.0)	4 (8.3)	0.069
Type 2 diabetes mellitus (%)	51 (19.2)	53 (22.6)	0.360	5 (25.0)	13 (27.1)	0.860
Left atrial appendage thrombi (%)	25 (10.5)	10 (4.7)	0.025	–	–	
Post-infarction aneurysm (%)	45 (16.9)	30 (12.8)	0.187	4 (20.0)	8 (16.7)	0.743
Heart failure (New York Heart Association)						
Class I	84 (31.6)	70 (29.9)	0.687	6 (30.0)	9 (18.8)	0.308
Class II	129 (48.5)	111 (47.4)	0.813	10 (50.0)	26 (54.2)	0.754
Class III	53 (19.9)	53 (22.6)	0.457	4 (20.0)	13 (27.1)	0.539
Class IV	–	–	0.457	–	–	–

BMI, body mass index.

Analysis of SCAG results had shown that single-vessel coronary disease was detected more often among indigenous males, as well as coronary stenosis with 50–75% and 75–90% of constriction; while multiple-vessel coronary stenosis was detected more often among non-indigenous males as well as stenosis with more than 90% constriction. Average number of diseased arteries was 2.1±0.1 vs. 2.4±0.1; p=0.001, respectively (Table III).

**Table III:** Incidence and stages of stenosis (coronary artery disease) among Yakutia residents (n in %)

	Incidence of stenosis in various stages					
	
	Males			Females		
			
Stages of coronary stenosis	I n=266 (%)	NI n=234 (%)	p	I n=20 (%)	NI n=48 (%)	p
<50%	33 (12.5)	28 (12)	0.880	2 (10.0)	6 (12.5)	0.771
50–75%	48 (18.0)	27 (11.5)	0.042	7 (35.0)	8 (16.7)	0.101
75–90%	76 (28.6)	52 (22.2)	0.105	4 (20.0)	14 (29.2)	0.437
>90%	109 (40.9)	127 (54.3)	0.003	7 (35.0)	20 (41.6)	0.610

Total calcium scores based on multi-detector CT results were found to be significantly lower among indigenous males than among non-indigenous males: 349.1±129.8 vs. 621.8±115.2 units, respectively (p=0.011).

All voltage signs of LV hypertrophy among males were detected more often among indigenous patients (Table IV). LV hypertrophy with the presence of one or more voltage signs was found in 55% of indigenous males and in 29% of non-indigenous males (p=0.001).

**Table IV:** Incidence of left ventricular hypertrophy in patients with coronary atherosclerosis resining in Yakutia (by echocardiographic findings)

	Males, n (%)			Females, n (%)		
		
Voltage signs of LV hypertrophy	I n=200 (%)	NI n=174 (%)	p	I (n=16)	NI (n=42)	p
R_aVL_ >11 mm	54 (27.0)	16 (9.2)	0.001	3 (18.8)	6 (14.3)	0.675
Cornell voltage	38 (19.0)	14 (8.0)	0.002	5 (31.3)	10 (23.8)	0.563
Cornell product	76 (38.0)	34 (19.5)	0.001	7 (43.8)	12 (28.6)	0.271
Sokolow-Lyon index	36 (18.0)	18 (10.3)	0.036	3 (18.8)	8 (19.0)	0.979
R_V5(V6)_ ≥27 mm	28 (14.0)	14 (8.0)	0.069	2 (12.5)	2 (4.8)	0.299
Gubner-Ungerleider index (R_I_+S_III_)	26 (13.0)	6 (3.4)	0.001	2 (12.5)	2 (4.8)	0.299

Based on the results of Holter ECG monitoring, episodes of painless ischaemia occurred more often among indigenous males compared to painful ischaemia episodes (63 vs. 38%; p=0.028).

Among males all cardiac structure–function indicators were found to be significantly higher among indigenous patients then non-indigenous (Table V). Eccentric type LV hypertrophy was detected more commonly among indigenous versus non-indigenous males (64% vs. 47%; p=0.001).

**Table V:** Mean echocardiographic values in patients with coronary atherosclerosis resining in Yakutia, M±m

	Indigenous		Non-indigenous		
		
Indicator	M±m	95% CI	M±m	95% CI	p
Males					
	n=236		n=214		
LAD index (sm/m^2^)	2.05±0.03	1.99–2.09	1.93±0.02	1.89–1.97	0.002
SWT index (sm/m^2^)	0.62±0.01	0.59–0.64	0.57±0.01	0.56–0.59	0.002
LVPWT index (sm/m^2^)	0.64±0.01	0.63–0.66	0.59±0.01	0.58–0.61	0.001
IDs index (sm/m^2^)	2.03±0.03	1.97–2.09	1.88±0.29	1.82–1.94	0.001
IDd index (sm/m^2^)	3.01±0.03	2.95–3.07	2.79±0.29	2.73–2.85	0.001
LVMM index (g/m^2^)	141.0±2.4	136.2–145.8	132.6±2.8	127.1–138.0	0.003
EDV index (mL/m^2^)	82.2±1.7	78.7–85.6	76.7±1.7	73.3–80.1	0.015
ESV index (mL/m^2^)	34.2±1.3	31.6–36.7	31.8±1.2	29.4–34.2	0.113
SI (mL/m^2^)	47.1±0.8	45.5–48.7	43.8±0.6	42.6–44.9	0.002
CI (L/min/m^2^)	3.0±0.1	2.9–3.2	3.0±0.1	2.9–3.2	0.147
LV hypertrophy, eccentric	70 (34.7)		40 (21.2)		0.003
LV hypertrophy, concentric	60 (29.7)		48 (25.4)		0.342
Normal geometry	62 (30.6)		75 (39.6)		0.063
Concentric remodeling	10 (5.0)		26 (13.8)		0.003
EF%	59.7±0.7	58.3–61.1	59.4±0.8	57.8–61.1	0.723
Females					
	n=20		n=48		p_I-NI_
LAD index (sm/m^2^)	2.34±0.09	2.11–2.38	2.26±0.08	2.11–2.30	0.443
SWT index (sm/m^2^)	0.65±0.21	0.62–0.72	0.59±0.28	0.61–0.69	0.360
LVPWT index (sm/m^2^)	0.63±0.02	0.60–0.71	0.58±0.04	0.60–0.67	0.706
IDs index (sm/m^2^)	2.09±0.10	1.97–2.36	2.09±0.09	1.88–2.29	0.178
IDd index (sm/m^2^)	3.05±0.11	2.93–3.32	3.05±0.08	2.81–3.06	0.106
LVMM index (g/m^2^)	130.6±7.5	113.8–145.8	110.3±8.7	113.1–140.7	0.459
EDV index (mL/m^2^)	72.5±1.4	69.5–75.6	66.7±2.8	61.1–72.4	0.187
ESV index (mL/m^2^)	32.8±2.4	27.7–37.9	31.5±2.7	26.1–36.8	0.225
SI (mL/m^2^)	44.4±1.8	40.7–48.2	42.8±1.2	40.4–45.1	0.757
CI (L/min/m^2^)	2.7±0.1	2.5–2.9	2.6±0.1	2.4–2.9	0.240
EF%	61.5±2.3	56.9–66.3	59.8±1.4	56.9–62.5	0.492

LAD, left atrial dimension; SWT, interventricular septal thickness; LVPWT, left ventricular posterior wall thickness; IDs, internal end-systolic dimension; IDd, internal end-diastolic dimension; LVMM, left-ventricular myocardial mass; ESV, end-systolic volume; EDV, end-diastolic volume; SI, stroke index; CI, cardiac index; EF, ejection fraction.

Laboratory results showed that triglyceride levels were significantly lower among indigenous males, however, platelet count was significantly higher among indigenous patients, both men and women, compared to non-indigenous patients (Table VI).

**Table VI:** Mean laboratory findings in patients, who had ischaemic heart disease with coronary atherosclerosis, residing in Yakutia

	Males, M±m			Females, M±m		
		
Indicator	I (n=266)	NI (n=234)	p	I (n=20)	NI (n=48)	p
Leucocytes (10^9^/L)	6.1±0.2	6.4±0.2	0.090	5.8±0.6	5.7±0.4	0.884
Platelets (10^9^/L)	216.4±5.8	185.8±4.7	0.001	275.1±43.6	200.3±12.5	0.050
ESR (mmHg)	13.2±0.9	12.3±1.0	0.404	23.6±3.0	20.3±3.5	0.403
Glucose level (mmol/L)	5.82±0.14	5.81±0.13	0.198	5.6±0.4	6.4±0.5	0.575
Fibrinogen level (g/L)	3.45±0.09	3.56±0.11	0.214	4.02±0.52	3.9±0.29	0.770
Total cholesterol (mmol/L)	4.8±0.1	4.9±0.1	0.910	5.2±0.3	5.5±0.2	0.169
HDL (mmol/L)	0.97±0.03	0.90±0.03	0.323	0.90±0.02	0.95±0.01	0.358
LDL (mmol/L)	3.12±0.06	3.22±0.09	0.572	3.32±0.26	3.55±0.12	0.178
Triglycerides (mmol/L)	1.45±0.03	1.56±0.04	0.030	1.53±0.08	1.64±0.06	0.106

ESR, erythrocyte sedimentation rate; HDL, high-density lipoprotein; LDL, low-density lipoprotein.

## Discussion

Less severe coronary artery disease among indigenous population correlated with the previous epidemiological studies conducted among healthy population in Yakutia. Possible explanation of similar frequencies of myocardial infarctions among indigenous and non-indigenous patients with presence of less severe atherosclerosis among the indigenous population could be the following: acute coronary events among the indigenous population were associated with thrombi (clot) and with high platelet count rather than with the severity of coronary stenosis. Predisposition to thrombi development among the indigenous population may be suggested, but this must be further investigated.

High incidences of painless ischaemia and of myocardial infarctions without angina pectoris in the indigenous population had been noted by other researchers from Yakutia, but reasons for this had remained incompletely understood (8). Personal characteristics, social status and cultural characteristics had an important role to play.

The observed high incidence of LV hypertrophy and relatively enlarged heart chambers in indigenous patients correlated with population studies conducted in Chukotka. Based on echocardiographic findings in those studies, LV hypertrophy had been detected 2–2.5 times more often in a group of indigenous population of coastal villages than among indigenous population of an urban area, Novosibirsk city (9). High incidence of arterial hypertension among indigenous males can be named as one of the reasons for LV hypertrophy (10). It should be noted, that arterial hypertension was also thought to be a typical “adaptation disease”. And the patterns, in which such diseases progress, depended on many factors: climatic, ecological, heliophysical and psychosocial factors (9–12).

## Conclusions

- In the Sakha Republic (Yakutia) single-vessel coronary disease, coronary stenosis with 50–75% and 75–90% of constriction were detected more often among indigenous males, while multiple-vessel coronary stenosis was detected more often among non-indigenous males as well as stenosis with more than 90% of constriction. Lower calcium score mean (349.1±129.8 vs. 621.8±115.2) was observed among indigenous patients compared to non-indigenous patients;
- Painless myocardial infarction, painless ischaemia, arterial hypertension, AF were detected more often among indigenous male compared to non-indigenous males;
- Based on the results of ECG and echocardiographic examinations, LV hypertrophy, particular eccentric type of hypertrophy, was found more commonly among indigenous than non-indigenous participants; and
- By laboratory findings, indigenous men had significantly lower triglyceride levels, while platelet counts were higher compared to non-indigenous patients. Obesity was observed less frequent among indigenous men compared to non-indigenous men.

## Limitations

This study was conducted without regard to patient compliance, alcohol consumption and physical activity. It must be noted, that indigenous patients were mostly from regions with highly unfavorable access to medical services. The study results regarding women were not very reliable due to small sample size. The differences observed in this study are disputable and call for further studies as well as the collection of reliable data for women. No multivariate analysis was done. All these limitations could have influence on the results.
